# Characteristics of oral microbiome of healthcare workers in different clinical scenarios: a cross-sectional analysis

**DOI:** 10.1186/s12903-022-02501-x

**Published:** 2022-11-10

**Authors:** Zhixia Zhang, Wenyi Yu, Guangyao Li, Yukun He, Zhiming Shi, Jing Wu, Xinqian Ma, Yu Zhu, Lili Zhao, Siqin Liu, Yue Wei, Jianbo Xue, Shuming Guo, Zhancheng Gao

**Affiliations:** 1 Nursing Department, Linfen Central Hospital, 041000 Shanxi, Shanxi China; 2 Department of Respiratory and Critical Care Medicine, Peking University People’s Hospital, Beijing, China; 3 Science and Education Department, Linfen Central Hospital, Hainan, Shanxi China; 4 Cardiology Department, Linfen Central Hospital, Hainan, Shanxi China; 5 The Stomatology College of Binzhou Medical University, Yantai, Shandong China; 6 Nursing College of Shanxi Medical University, Shanxi, China; 7 Department of Pulmonary and Critical Care Medicine, Peking University People’s Hospital, 100044 Beijing, China

**Keywords:** Healthcare institutes, Coronary care unit, Healthcare worker, 16S rRNA, Oral microbiome, Microbial alteration

## Abstract

The environment of healthcare institutes (HCIs) potentially affects the internal microecology of medical workers, which is reflected not only in the well-studied gut microbiome but also in the more susceptible oral microbiome. We conducted a prospective cross-sectional cohort study in four hospital departments in Central China. Oropharyngeal swabs from 65 healthcare workers were collected and analyzed using 16S rRNA gene amplicon sequencing. The oral microbiome of healthcare workers exhibited prominent deviations in diversity, microbial structure, and predicted function. The coronary care unit (CCU) samples exhibited robust features and stability, with significantly higher abundances of genera such as *Haemophilus*, *Fusobacterium*, and *Streptococcus*, and a lower abundance of *Prevotella*. Functional prediction analysis showed that vitamin, nucleotide, and amino acid metabolisms were significantly different among the four departments. The CCU group was at a potential risk of developing periodontal disease owing to the increased abundance of *F. nucleatum.* Additionally, oral microbial diversification of healthcare workers was related to seniority. We described the oral microbiome profile of healthcare workers in different clinical scenarios and demonstrated that community diversity, structure, and potential functions differed markedly among departments. Intense modulation of the oral microbiome of healthcare workers occurs because of their original departments, especially in the CCU.

**Supplementary Information:**

The online version contains supplementary material available at 10.1186/s12903-022-02501-x.

## Introduction

Unique conditions render the microbial composition in healthcare institutes (HCIs) vastly different from that in the external natural environment [1–3]. Hospitalized individuals are at a greater risk of human-related microorganisms or pathogens colonizing their nasal cavity compared with non-hospitalized groups [4]. Additionally, the diversity and compositions of the microbiome change dynamically in different clinical scenarios, which may be correlated with different indoor environmental conditions [5]. A previous study reported that the gut microbiome of intensive care unit (ICU) workers, compared with non-ICU workers, showed a significantly increased abundance of *Dialister*, *Enterobacteriaceae*, *Phascolarctobacterium*, *Pseudomonas*, *Veillonella*, and *Streptococcus* and a marked depletion of *Faecalibacterium*, *Blautia*, and *Coprococcus* [6]. Beyond the aspect of common colonizing microorganisms, potential pathogens in hospital settings cannot be neglected. Thus, different clinical scenarios in HCIs may affect the microbiota of healthcare workers. Microbial ecology in HCIs comprehensively affects the health of healthcare workers. Studies have shown that the adverse microbial characteristics of HCIs could increase the incidence of microbial infections in staff [7–9]. Therefore, identifying the microbial status of healthcare workers is of extreme importance in the control of nosocomial infections.

Previous studies have widely focused on stool samples from healthcare workers to elucidate gut microbial properties, while respiratory samples are rarely involved. The respiratory tract is a common route of nosocomial infections [10], and the occurrence and progression of many diseases have been related to alterations in the respiratory microbiome [11–13]. The oral microbiome closely resembles that of the lung [14]. Numerous empirical studies have illustrated the dominance of Firmicutes, Actinobacteria, Proteobacteria, Fusobacteria, Bacteroidetes, and Spirochaetes in a healthy oral cavity, constituting 96% of the total oral bacteria [15, 16]. It has been noted that specific genera or species play roles in oral health and disease, even extraoral sites in systemic diseases [17]. Hence, exploring the extent of the oral microbiome through non-invasive operations may reflect more internal respiratory tract characterizations [18, 19]. Meanwhile, 16 S rRNA gene sequences have potential advantages for detecting the oral microbiome, whether they are cultivated or not [17].

Currently, the composition of the oral microbiome of healthcare workers and the influence of the HCI environment remain unclear. Here, we profiled the microbial community of oropharyngeal swabs from healthcare workers in different clinical scenarios in a hospital based on 16 S rRNA gene sequencing targeting multiple bacterial hypervariable regions. Oral microbial composition and predicted functional characteristics were analyzed to evaluate the impact of the hospital environment on the oral microbiome of healthcare workers.

## Methods

### Participants and sample collection

A total of 65 full-time healthcare workers from coronary care unit (CCU, n = 12), ICU (n = 16), operating room (OR, n = 16) and department of respiratory medicine (RES, n = 21) of Linfen Central Hospital (Shanxi Province, China) were recruited. Subjects were excluded if they had a respiratory tract infection or respiratory tract disease, or were treated with antibiotics in three months prior to sampling, or had worked less than one year in the hospital. Participants were asked to avoid eating and drinking for three hours prior to sampling. All fresh oropharyngeal swabs were collected by one operator within two hours and immediately stored at −80 °C until DNA extraction. Additional information on age, gender, position, seniority, sleeping and dietary habits were obtained through questionnaires.

### DNA preparation and sequencing

Total genomic DNA was extracted from oropharyngeal swabs using a TIANamp Micro DNA Kit (Tiangen, China) following the manufacturer’s instructions. We amplified the corresponding hypervariable regions (V2, V3, V4, V6-7, V8 and V9) of the 16 S rRNA with two primer pools in an Ion 16 S^™^ Metagenomics Kit (ThermoFisher Scientific, UK). A total of six primer pairs amplifying multiple hypervariable regions listed above were split into two pools to avoid possible interference during the amplification reaction. Every DNA template was amplified in two primer pools, and then two tubes of PCR products were combined to obtain complete amplification products from multiple hypervariable regions. XP beads were next used to purify the amplification products and quantified by Qubit4 (ThermoFisher Scientific, USA). Purified amplicons were ligated with barcodes to prepare the libraries. Then, libraries were pooled in equimolar amounts on chip 530 and sequenced to single-end, 250-base-pair reads on an Ion GeneStudio S5 System (ThermoFisher Scientific, USA) based on the Ion Reporter metagenomics workflow (Ion 16 S mNGS), which had 100% sensitivity when accounting for the genus level of detection [20]. All amplified regions were sequenced. Sequencing of multiple variable regions allows for higher resolution.

### Bioinformatic analyses

Quality filtering, trimming and dereplication of raw sequencing reads were conducted automatically on the Ion Reporter metagenomics workflow, relying on default parameters. We next used the UCHIME algorithm [21] to remove the chimeric sequences and used unoise3 [22] to generate denoising amplicon sequence variants (ASVs). Taxonomy assignment was performed based on vsearch [23] referring to the SILVA (V 138.1) [24] and GreenGene database [25] with a threshold of 97%. Reads with an alignment rate below 97% were rejected. We aligned reads from different variable regions with the bacterial reference genome separately. A consensus table was created by summing all read counts from different regions with identical taxonomic rank detection. We rarefied the sequencing data and then evaluated the alpha diversity by the Good’s average index, Chao1 index, abundance-based coverage estimator (ACE) index, Shannon index and Simpson index. Permutational multivariate analysis of variance (PERMANOVA) and analysis of similarity (ANOSIM) based on the Bray‒Curtis distance were used to evaluate the beta diversity.

Differential bacterial taxa among groups were obtained using linear discriminant analysis effect size (LEfSe) [26] with the criteria of LDA > 2 (or LDA > 4) and *P* < 0.05. Microbiome phenotypes were predicted using BugBase [27]. PICRUSt2 [28] was used to identify microbiome-associated pathways from the inferred metagenomes of taxa using the ‘stratified’ option. We applied the Pearson correlation algorithm to identify associations across bacterial genera, representing correlation strength and assigned them to the edges. For the Pearson correlation table, we used the cytoHubba [29] plugin in Cytoscape [30] (V 3.9.1) to find Hubba nodes based on the maximum cross-correlation algorithm. The Hubba nodes represent the taxonomy that had the highest correlation with the other genera. Then, we took the intersection of correlation nodes and the top 10 Hubba nodes and retained nodes whose absolute correlation value was greater than 0.6.

### Statistical analysis

Parametric continuous variables are presented as mean ± standard deviation, and abnormally distributed continuous variables are presented as medians and interquartile range (25th and 75th percentiles). Categorical variables are described as numbers (percentages). Student’s *t*-test and analysis of variance (ANOVA) with post hoc Tukey HSD test were used to compare continuous parametric data conforming to normal distribution. Abnormally distributed continuous variables were compared using the Mann‒Whitney U test or Kruskal‒Wallis H-test. Categorical variables were analyzed using the chi-square or Fisher’s test. All tests were two-sided. P values were corrected using FDR, and *P* < 0.05 was considered statistically significant [31]. We performed statistical analyses by using SPSS version 25 software.

## Results

### Demographic characteristics of the study population

We investigated the demographics of the participants, including several indicators that may affect the oral microbiome [32, 33]. The statistical results are presented in Table 1. The mean age was 32.03 years old, and 76.9% were female. A total of 58.5% of the participants were nursing staff, and the remainder were resident doctors. The mean seniority was 7.86 years. The median number of sleeping hours per day and intake times of sweets/desserts per week were 6.50 and 3.00, respectively. There were no significant differences in these parameters among the CCU, ICU, OR, and RES groups. All participants were free of metabolic diseases such as diabetes and hyperlipidemia.

**Table 1:** Baseline characteristics of participants

Characteristics	Total (n = 65)	CCU (n = 12, 18.5%)	ICU (n = 16, 24.6%)	OR (n = 16, 24.6%)	RES (n = 21, 32.3%)	P value
Age	32.03 ± 4.786	28.75 ± 3.519	32.88 ± 4.440	32.31 ± 5.326	33.05 ± 4.717	0.064^a^
Sex						0.103^b^
Male	15 (23.1%)	1 (8.3%)	4 (25%)	7 (43.75%)	3 (14.3%)	
Female	50 (76.9%)	11 (91.7%)	12 (75%)	9 (56.25%)	18 (85.7%)	
Position						0.151^b^
Doctor	27 (41.5%)	2 (16.7%)	7 (43.75%)	6 (37.5%)	12 (57.1%)	
Nurse	38 (58.5%)	10 (83.3%)	9 (56.25%)	10 (62.5%)	9 (42.9%)	
Seniority	7.86 ± 4.680	5.50 ± 2.680	9.06 ± 3.890	8.31 ± 4.895	7.95 ± 5.670	0.237^a^
Sleeping hours per day	6.50 (5.75–7.50)	6.92 ± 0.70	6.34 ± 1.179	6.50 ± 1.140	6.26 ± 1.300	0.441^c^
Intake times of sweets/desserts per week	3.00 (1.00–4.00)	2.42 ± 1.832	2.75 ± 1.880	2.88 ± 1.996	2.86 ± 2.056	0.924^a^

P values were calculated using a, ANOVA; b, chi-square test; and c. Kruskal‒Wallis test

### Distinct oral microbial structure and diversity among departments

The rarefaction curves of all samples demonstrated that the sequencing coverage was sufficient to represent the microbial composition (Supplementary Material Fig. S1). A total of 13,251 ASVs were identified in all 65 samples. After filtering out ASVs found in less than two samples, 1519 ASVs were reserved for downstream analysis with a minimum relative abundance of 0.05% [34].

**Fig. 1:**
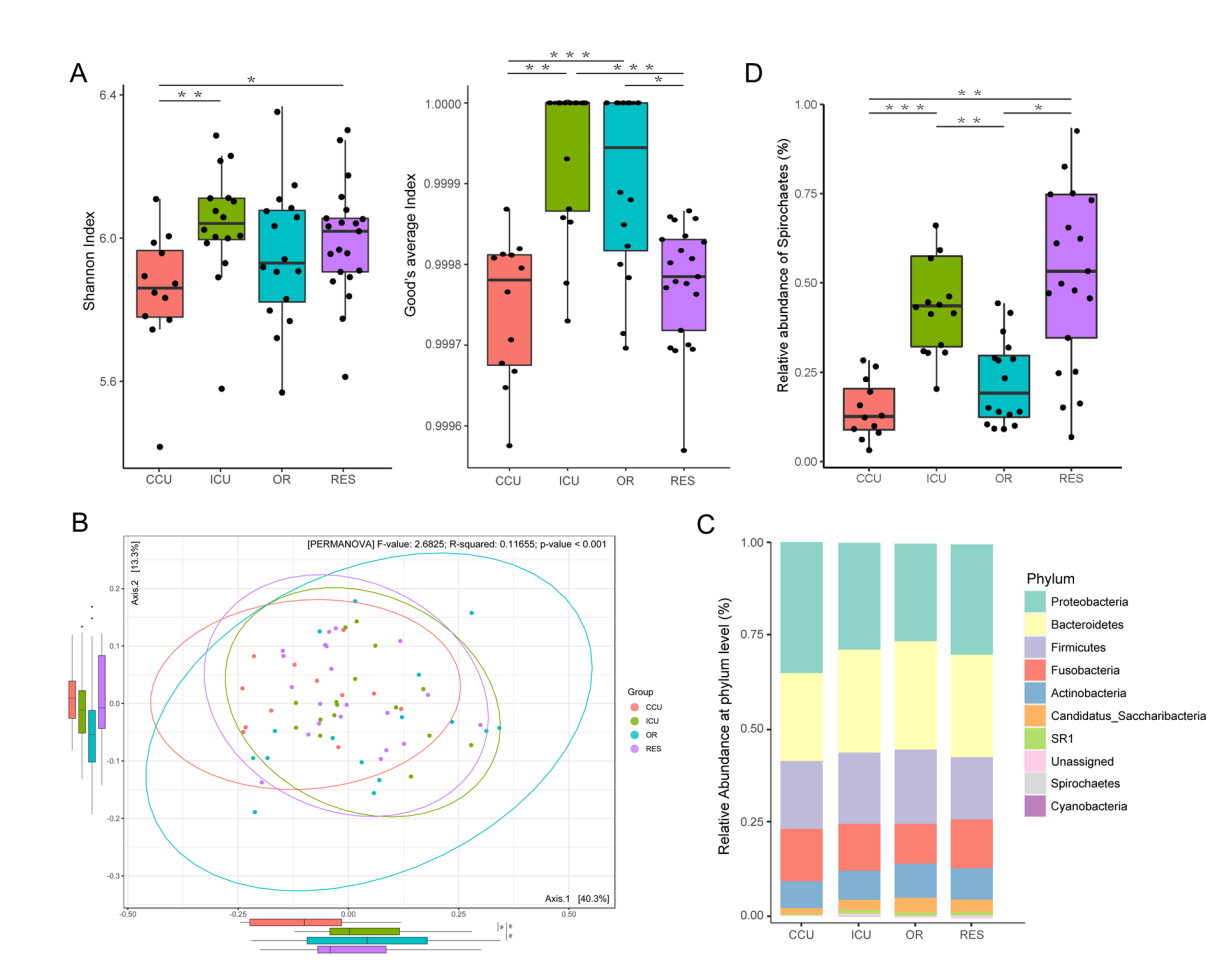
Structure and alteration of the oral microbiome in healthcare workers from different departments (A) Alpha diversity indices, including the Shannon index (left, ANOVA test, CCU versus ICU *P* = 0.007, CCU versus RES *P* = 0.007) and Good’s average index (right, Kruskal‒Wallis test, CCU versus ICU *P* = 0.000, CCU versus OR *P* = 0.005, ICU versus RES *P* = 0.000, OR versus RES *P* = 0.011), represent differences in within-sample diversity among departments. (B) PCoA plot showing significant deviation among departments based on PERMANOVA at the genus level. Box plots of the primary (down) and second (left) constrained axes are shown. (C) Stacked bar chart showing the relative abundance of departments at the phylum level. (D) Box plot revealing significant deviation in the relative abundance of Spirochaetes. * *P* < 0.05; ** *P* < 0.01; *** *P* < 0.001

We first assessed alpha diversity within individuals. The CCU group showed the lowest level of microbial diversity compared to the other groups, which was reflected in the Shannon and Good’s coverage index (Fig. 1A, Supplementary Material Fig. S2). In terms of beta diversity, PERMANOVA based on Bray–Curtis distance hardly exhibited obvious intergroup clustering of microbiota structure, even if statistically significant (Fig. 1B). Furthermore, ANOSIM analysis for pairwise comparison showed that the bacterial community of the CCU provided the most significant between-group differential components (Supplementary Material Fig. S3). We also tested whether the oral microbiome of healthcare workers is associated with other demographic characteristics. The department proved to be the main grouping factor accounting for the variance of the oral microbiota compared to other confounders, such as age, sex, position, diet, and sleep, although there was also a significant difference between those with 5–10 years of seniority and those with more than 10 years of seniority (Supplementary Material Fig. S3, *P* = 0.027). Taken together, these findings revealed prominent differences in oral microbial structure and diversity among the departments.

### Differences in the oral microbiome between hospital departments

**Fig. 2:**
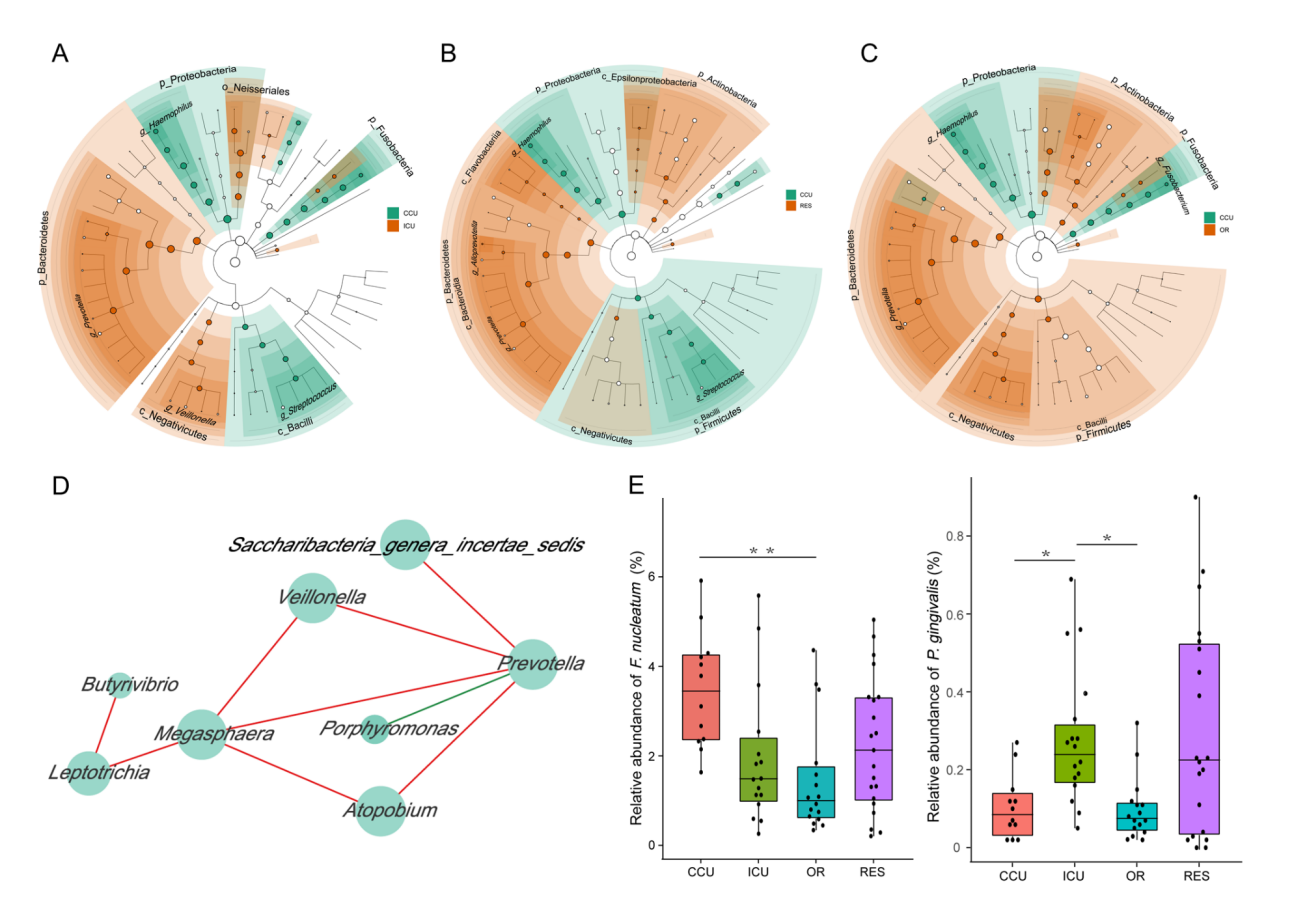
Department-associated microbial taxa identified via LEfSe analysis Cladogram plots showing the significant differences in relative abundance (LDA > 4) between ICU (A), RES (B) and OR (C) versus CCU. (D) Co-occurrence networks showing associations among significant oral microbial taxa based on Pearson correlation and maximum cross correlation algorithm. The size of the nodes indicates their degree of hub. Connecting lines represent the Pearson correlation coefficient ρ > 0.6 (red line) or < -0.6 (green line). (E) Box plot showing the relative abundance of *F. nucleatum* (CCU versus OR, *P = 0.002*) and *P. gingivalis* (CCU versus ICU, *P =* 0.04; OR versus ICU, *P =* 0.016) across departments. The Kruskal‒Wallis test was used

The microbiome profile comprised 13 phyla, 20 classes, 29 orders, 53 families, and 97 genera. Core phyla were defined as those identified in all samples. Nine core phyla, Bacteroidetes, Firmicutes, Proteobacteria, Actinobacteria, Fusobacteria, Candidatus Saccharibacteria, Spirochaetes, SR1, and Synergistetes, are shown in Fig. 1C. We conducted the Kruskal–Wallis test to compare the relative abundance of the nine core phyla across groups, and Spirochaetes was significant (Fig. 1D, CCU versus ICU *P* = 0.000; CCU versus RES *P* = 0.001; ICU versus OR *P* = 0.005; OR versus RES *P* = 0.043). We further conducted LEfSe analysis to identify significant differences in abundance between departments, considering CCU as the basic group. We identified 66 microbial taxa (19 CCU-enriched versus 47 ICU-enriched) that differed significantly in relative abundance between CCU and ICU, 36 microbial taxa (14 CCU-enriched versus 22 OR-enriched) that differed significantly in relative abundance between CCU and OR, and 40 microbial taxa (10 CCU-enriched versus 30 RES-enriched) that differed significantly in relative abundance between CCU and RES (Supplementary Table S1). To identify more reliable differential taxa, we set a stricter filter with LDA > 4. The relative abundance of *Haemophilus* in the CCU group was higher than that in the other groups, while *Prevotella* showed the opposite trend. Moreover, compared with ICU and RES workers, CCU workers showed a higher abundance of taxa belonging to Fusobacteria (*Fusobacterium*) and Firmicutes (*Streptococcus*). The relative abundance of Bacilli, a class belonging to Firmicutes, in the CCU suggested a level of depletion to the OR (Fig. 2A-C, Supplementary Material Fig. S4).

We further evaluated the effects of various grouping characteristics on the oral microbiome. Factors such as age, sex, position, diet, and sleep could not be used to distinguish the differential microbiota. Notably, when we grouped the participants by seniority, several differential bacterial genera were identified (Supplementary Material Fig. S5), suggesting that oral microbial diversification of healthcare workers is related to seniority.

We next performed co-occurrence network analysis and found vital interconnections within the oral microbiome, indicating that these healthcare-worker-altered taxa did not occur independently in the oral environment. *Megasphaera, Prevotella, Leptotrichia, Atopobium*, and *Veillonella* may be essential genera that shape the oral microbiome of healthcare workers, accompanied by rich multivariate interrelationships and strong correlations between each other (Fig. 2D). *Porphyromonas* was negatively correlated with *Prevotella*, whereas the rest were positively correlated.

### Differences in periodontal pathogens

We further explored the distribution of critical periodontal pathogens among departments [35–40]. The relative abundance of *F. nucleatum* increased significantly in the CCU group compared to the OR group, but compared to the ICU group the increase was not significant (Fig. 2E). The relative abundance of *P. gingivalis* in the ICU was higher than that in the OR or CCU. However, the relative abundance of *P. gingivalis* was approximately 10 times lower than that of *F. nucleatum* (Supplementary Material Fig. S6). These results suggest a potential risk of periodontal disease in the CCU and the ICU.

### Potential function of the oral microbiome

We analyzed the predicted phenotypes based on taxonomic classification using BugBase. BugBase categorized six main bacterial phenotype categories: Gram staining, oxygen tolerance, ability to form biofilms, mobile element content, pathogenicity, and oxidative stress tolerance. Phenotypes were inferred based on experimental data and pathway/gene presence information collected from various databases, such as Integrated Microbial Genomes (IMG) and the PathoSystems Resource Integration Center (PATRIC) [27]. BugBase data between departments were compared using pairwise Mann-Whitney-Wilcoxon test. Facultative anaerobic bacteria were more abundant in the CCU group than in the other groups (CCU versus ICU, *P* = 0.017; CCU versus OR, *P* = 0.047; CCU versus RES, *P* = 0.048; Supplementary Material Fig. S7A). The relative abundance of gram–positive bacteria in the CCU and OR groups was higher than that in the RES group (CCU versus RES, *P* = 0.000; OR versus RES, *P* = 0.043), whereas the opposite was true for gram–negative bacteria (CCU versus RES, *P* = 0.000; OR versus RES, *P* = 0.043; Supplementary Material Fig. S7B–C).

**Fig. 3:**
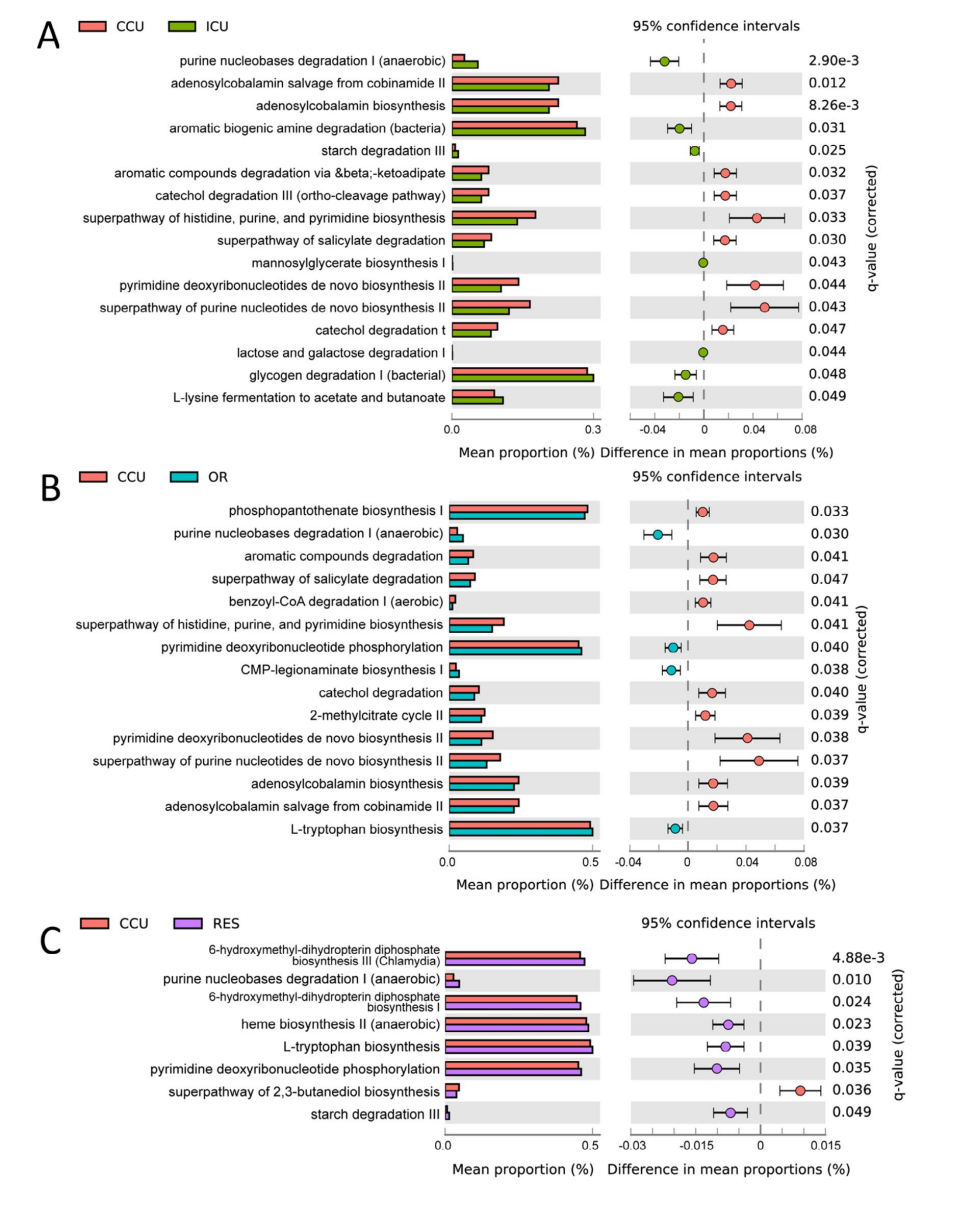
Functional characterization of different groups based on PICRUSt2 analysis Bar chart showing the functional difference (corrected q–value < 0.05) between ICU (A), OR (B) and RES (C) versus CCU. Data were compared by two-sided Welch’s t–test and filtered for false discoveries using the Benjamini-Hochberg method. Items with q-values less than 0.05 are shown in the figure. The above analyses were all performed on stamp software [52].

PICRUSt2 was employed to impute MetaCyc pathway abundance from the original taxonomic assignment. In total, 399 pathways were annotated. Metabolic pathway data were compared by two-sided Welch’s t–test and filtered for false discoveries using the Benjamini-Hochberg method. Items with q-values less than 0.05 were considered significant. Sixteen differential pathways were elucidated between CCU and ICU, two of which were responsible for nucleotide biosynthesis (“pyrimidine deoxyribonucleotides de novo biosynthesis II” and “superpathway of purine nucleotides de novo biosynthesis II”) and were enriched in CCU (Fig. 3A). In addition, vitamin B12 synthesis was also upregulated in CCU(“adenosylcobalamin biosynthesis from cobyrinate a,c-diamide I” and “adenosylcobalamin salvage from cobinamide II”) (Fig. 3A). We identified 15 differential pathways between the CCU and OR groups. Nucleotide and vitamin B12 biosynthesis processes (“superpathway of purine nucleotides de novo biosynthesis II,” “pyrimidine deoxyribonucleotides de novo biosynthesis II,” “adenosylcobalamin biosynthesis from cobyrinate a,c-diamide I,” and “adenosylcobalamin salvage from cobinamide II”) were more active in CCU (Fig. 3B). Finally, the functional catalog, including biosynthesis and degradation of nucleotides, amino acids and starch, appeared to be enriched in the RES group compared to that in the CCU group (Fig. 3C). These results suggest that the predicted microbial functions of vitamin, nucleotide and amino acid metabolism were significantly different between the departments.

## Discussion

Healthcare workers are exposed to hospital environments and are constantly in contact with infected patients during daily work. High-risk exposure to transmissible bacteria affects not only the microbiome of the skin surface, but also the respiratory and digestive tracts. To the best of our knowledge, no study has adequately described the characteristics of the oral microbiome in healthcare workers. We demonstrated that the bacterial community diversity, structure, and potential function of staff in the CCU, ICU, OR, and RES departments differed markedly.

Since late 2019/early 2020, the COVID-19 pandemic has led to general universal masking in healthcare settings. Our study reflects the differences in the oral microbiota composition of healthcare workers from different HCI environments during the pandemic. We selected a single-center hospital in Shanxi Province, China, for the study, which excluded the influence of diet, the living environment, and cultural background as much as possible [41–43]. It can be speculated that the original departments led to the different compositions of the oral microbial community. In the analysis of beta diversity, our data suggested that the CCU contributed the most significant between-group differences. In the subsequent comparison of oral microbial compositional differences and functional analysis, the CCU also exhibited robust features and stability. We inferred that the oral microbiome of the CCU healthcare workers received characteristic modulations from their departments. Thus, it was reasonable to consider the CCU as the basic object for comparison with other groups. Microbial distribution showed deviation among departments, with an increased abundance of Spirochaetes in the ICU and RES. Numerous empirical studies have shown that oral Spirochaetes cause damage to periodontal tissue by the direct effect of bacterial enzymes and cytotoxic products of bacterial metabolism [44–46].

It seems that the performance of *F. nucleatum* was more weighted than that of *P. gingivalis* because of its greater relative abundance. Taken together, these findings indicate that healthcare workers in different departments face specific risks of periodontal disease.

At the genus level, the CCU group showed significant differences compared to other groups, with an elevated abundance of *Haemophilus* and decreased abundance of *Prevotella*, demonstrating a possible impact on the oral microbiome of healthcare workers in different clinical scenarios. In general, exposure to *Haemophilus* is most common in the department of respiratory medicine [47, 48]. However, the prevalence of *Haemophilus* spp. in the CCU was higher in our study. It has been reported that *Haemophilus* accounts for most gram–negative bacilli causing infective endocarditis [49–51], which is usually treated in the CCU. In contrast, the relatively depleted abundance of *Haemophilus* in the RES may represent stricter protection for healthcare workers in the Department of Respiratory Medicine.

This study had several limitations. First, the study design was not longitudinal, and it lacked long-term tracking and analysis. Second, to explore the stabilizing effects, we selected enrollees working for over one year. More studies are needed to monitor oral microbial changes in short-term healthcare workers. Third, we did not clarify how nonbacterial microbiota (fungi, viruses, and archaea) contribute to the oral microbiome. Finally, despite the observation of different oral microbiomes among departments, we could neither decipher the causal relationships of the differences nor evaluate the influence of such differences on the health of the participants.

## Conclusion

In this study, we provide a profile of the oral microbiome of healthcare workers and highlight the essential role of the HCI environment. Workers in the CCU are more likely to exhibit inherent microbiological characteristics, such as reduced diversity, significantly differentiated genera, and higher potential for periodontal diseases. Our study provides a reference for further understanding of the oral microbiological characteristics of healthcare workers. In light of our results, we propose that continuous monitoring of the oral microbiome of healthcare workers in different clinical scenarios should be considered to improve health.

## Electronic supplementary material

Below is the link to the electronic supplementary material.

**Additional File 1:** Supplementary Table S1

**Additional File 2:** Supplementary Figure

## Data Availability

The datasets generated and analyzed during the current study are available in the NCBI repository (http://www.ncbi.nlm.nih.gov/bioproject/841067, BioProject ID PRJNA841067).

## Abbreviations

HCI: Healthcare institute
ASV: Amplicon sequence variant
CCU: Coronary care unit
ICU: Intensive care unit
OR: Operating room
RES: Department of respiratory medicine.
