# Correction: Characteristics of oral microbiome of healthcare workers in different clinical scenarios: a cross-sectional analysis

**DOI:** 10.1186/s12903-022-02667-4

**Published:** 2022-12-22

**Authors:** Zhixia Zhang, Wenyi Yu, Guangyao Li, Yukun He, Zhiming Shi, Jing Wu, Xinqian Ma, Yu Zhu, Lili Zhao, Siqin Liu, Yue Wei, Jianbo Xue, Shuming Guo, Zhancheng Gao

**Affiliations:** 1 Nursing Department, Linfen Central Hospital, Hainan, 041000 Shanxi China; 2 Department of Respiratory and Critical Care Medicine, Peking University People’s Hospital, Beijing, China; 3 Science and Education Department, Linfen Central Hospital, Hainan, Shanxi China; 4 Cardiology Department, Linfen Central Hospital, Hainan, Shanxi China; 5 The Stomatology College of Binzhou Medical University, Yantai, Shandong China; 6 Nursing College of Shanxi Medical University, Shanxi, China

**Correction to: BMC Oral Health (2022) 22:481** 10.1186/s12903-022-02501-x

In this article, the affiliation details for the author Zhancheng Gao were incorrectly given as “Department of Respiratory and Critical Care Medicine, Peking University People's Hospital, Beijing, China” and “Department of Pulmonary and Critical Care Medicine, Peking University People's Hospital, Beijing, 100044, China” but should have been only “Department of Respiratory and Critical Care Medicine, Peking University People's Hospital, Beijing, China”.

The revised affiliations are given below:

Zhixia Zhang^1^, Wenyi Yu^2^, Guangyao Li^3^, Yukun He^2^, Zhiming Shi^4^, Jing Wu^1^, Xinqian Ma^2^, Yu Zhu^3^, Lili Zhao^2^, Siqin Liu^5^, Yue Wei^6^, Jianbo Xue^2^, Shuming Guo^1*^ and Zhancheng Gao^2*^

^1^Nursing Department, Linfen Central Hospital, 041000 Shanxi, Shanxi, China

^2^Department of Respiratory and Critical Care Medicine, Peking University People’s Hospital, Beijing, China

^3^Science and Education Department, Linfen Central Hospital, Hainan, Shanxi, China

^4^Cardiology Department, Linfen Central Hospital, Hainan, Shanxi, China

^5^The Stomatology College of Binzhou Medical University, Yantai, Shandong, China

^6^Nursing College of Shanxi Medical University, Shanxi, China

