# Supplementary material for: Characteristics of oral microbiome of healthcare workers in different clinical scenarios: a cross-sectional analysis
Source: BMC Oral Health. 2022 Nov 10;22:481. doi: 10.1186/s12903-022-02501-x (PMC9648452; doi:10.1186/s12903-022-02501-x)
Supplement: Supplementary file 2 — Additional File 2: Supplementary Figure [file 12903_2022_2501_MOESM2_ESM.docx]

Supplementary Figures


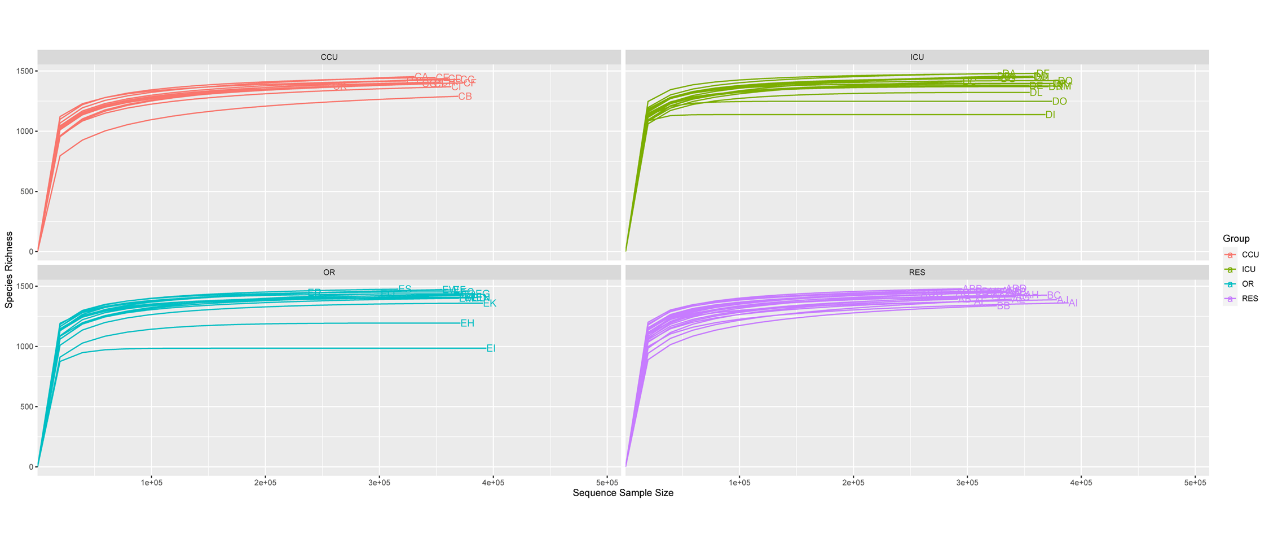


Fig. S1. Rarefaction curve indicating that the amount of sequencing reads per sample has reached saturation.


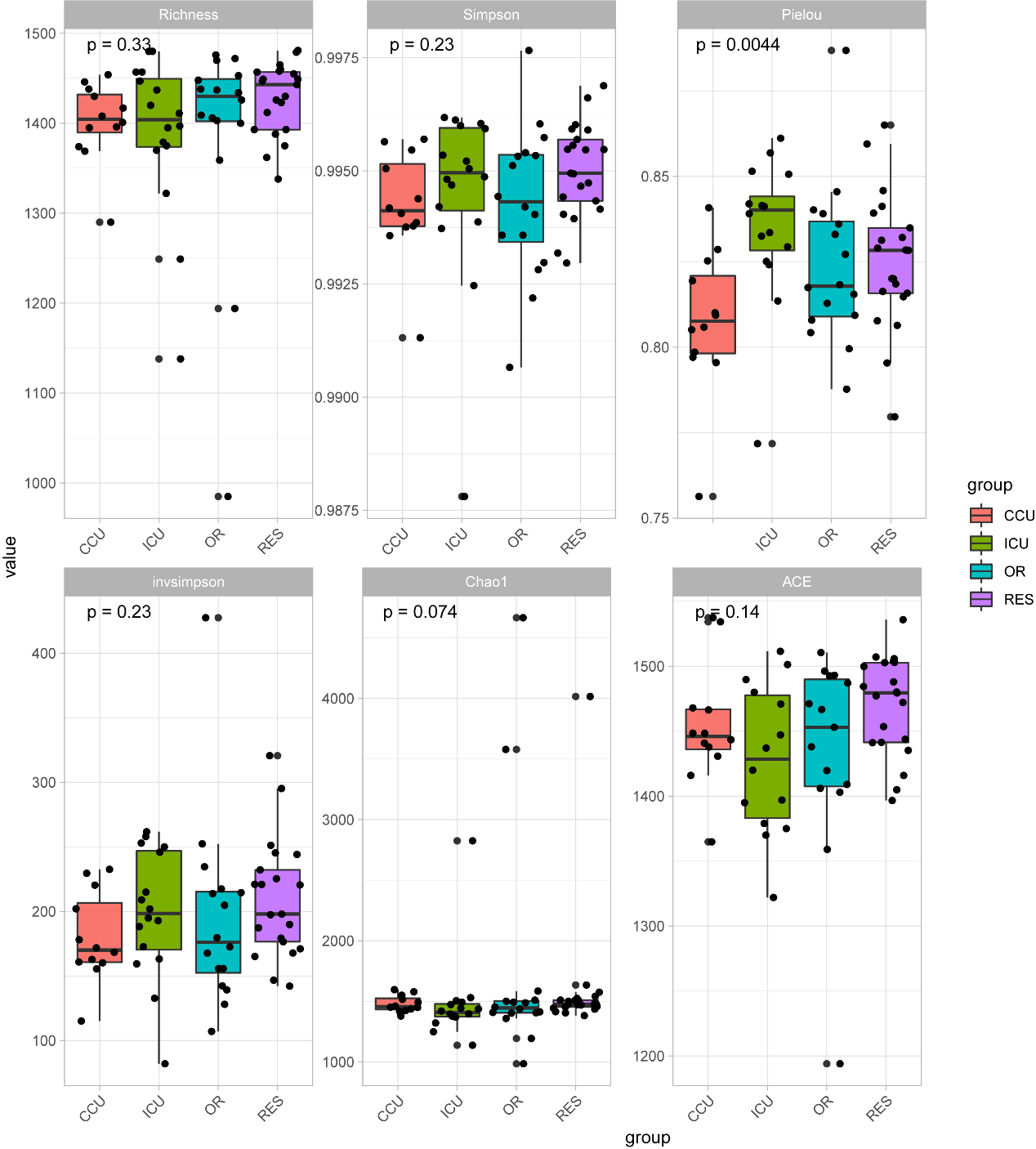


Fig. S2. All methods did not differ significantly between groups however Pielou was significant (p=0.0044).


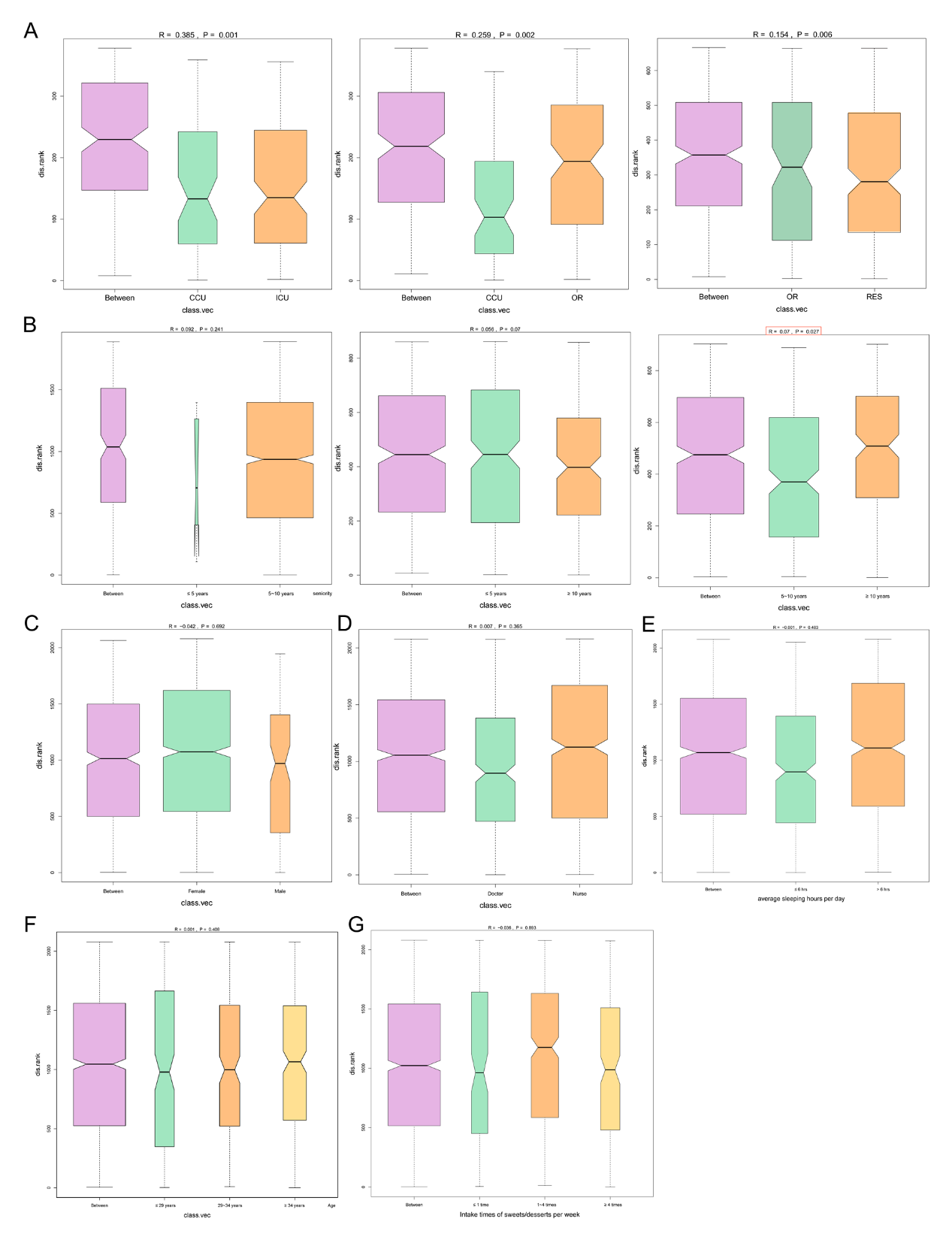


Fig. S3. (A) ANOSIM analysis evaluating the contribution to between-group differential microbial taxa of departments, and only statistically significant results are shown here (*P* < 0.05). ANOSIM analysis of the other grouping factors, including seniority (B), gender (C), position (D), sleep (E), age (F) and diet (G). Only department and seniority showed significant differences, respectively.


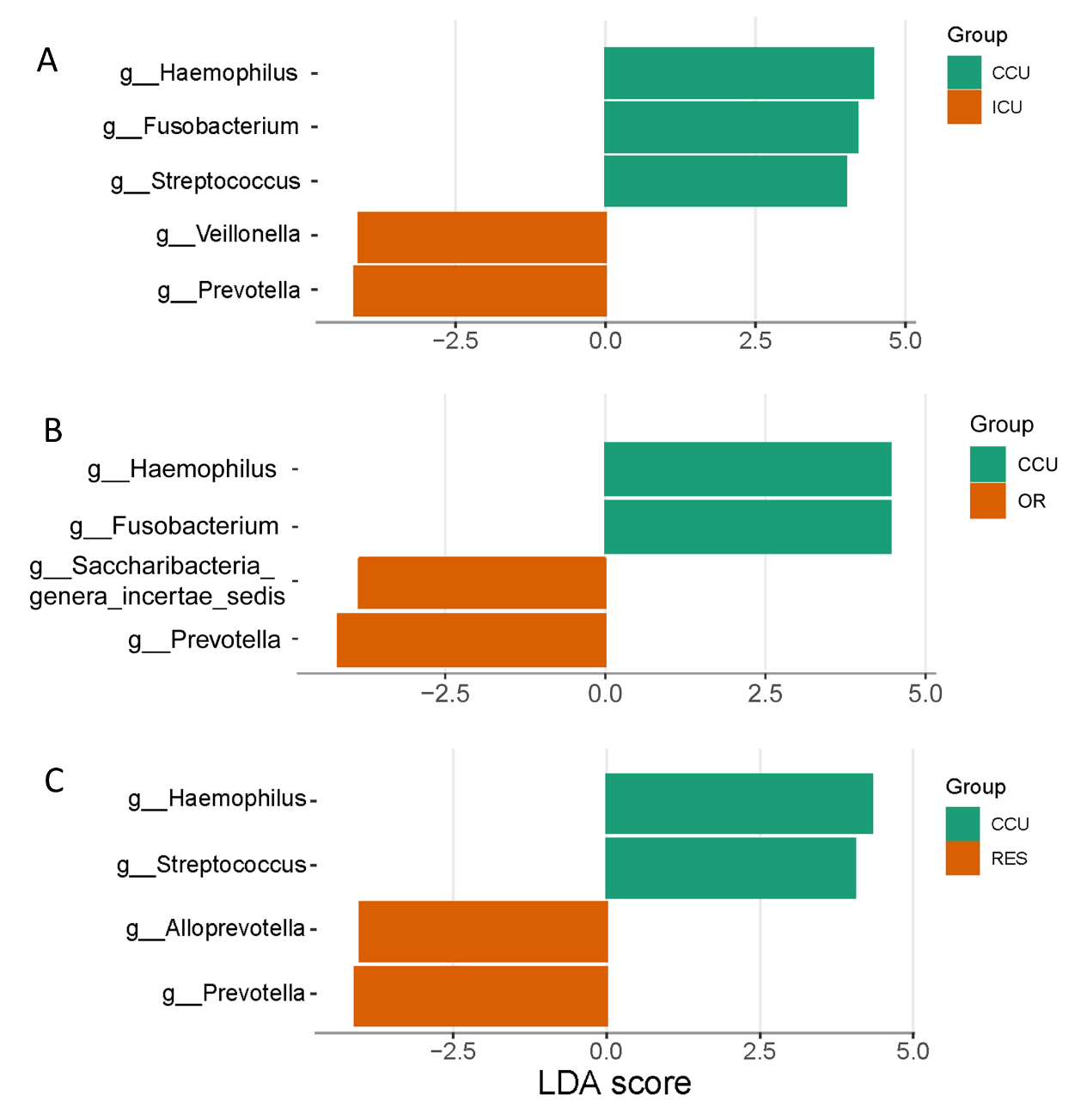


Fig. S4. Bar chart showing the significantly different genera between ICU (A), OR (B) and RES (C) versus CCU (LDA > 4).


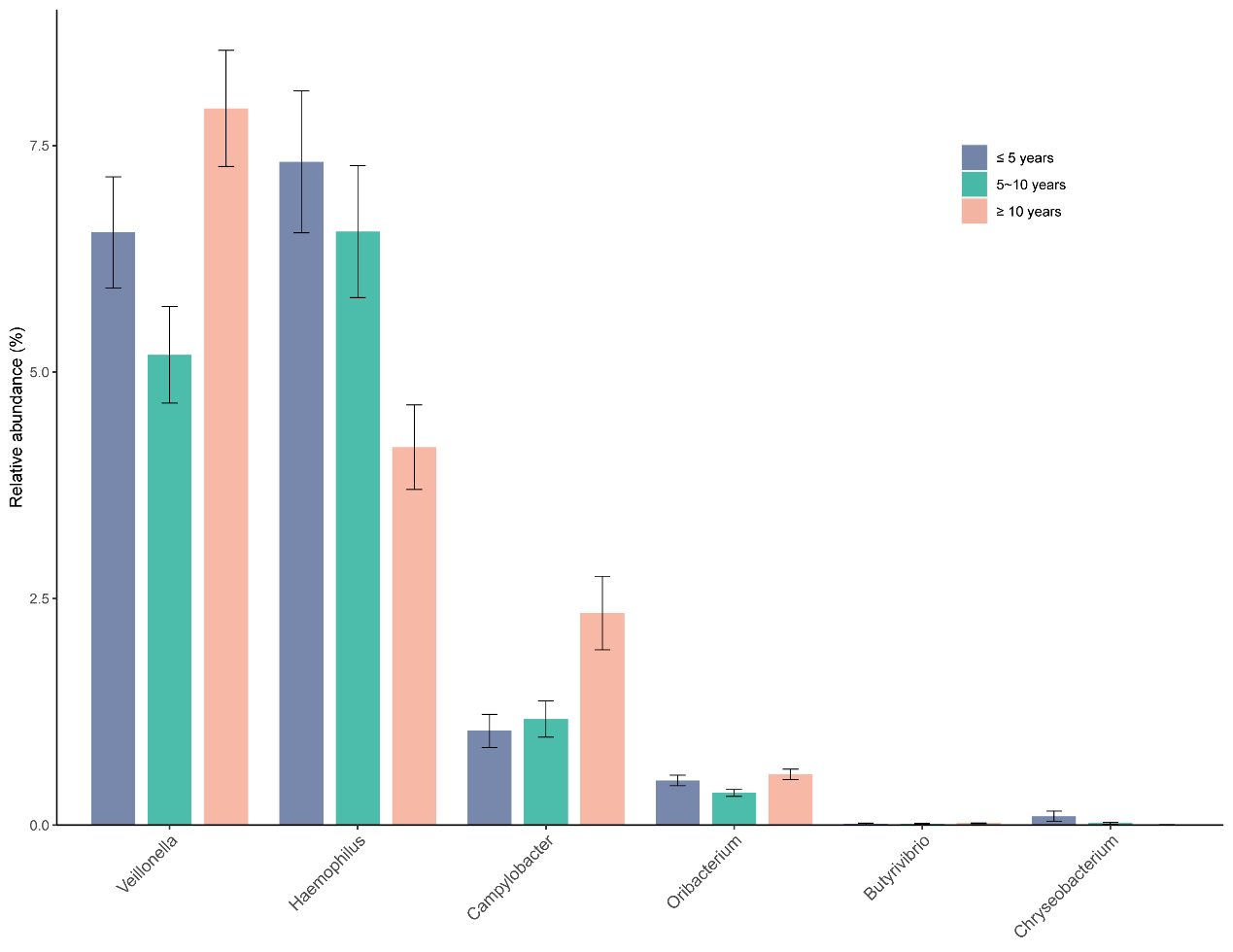


Fig. S5. Six differential genera were identified among seniority groups.


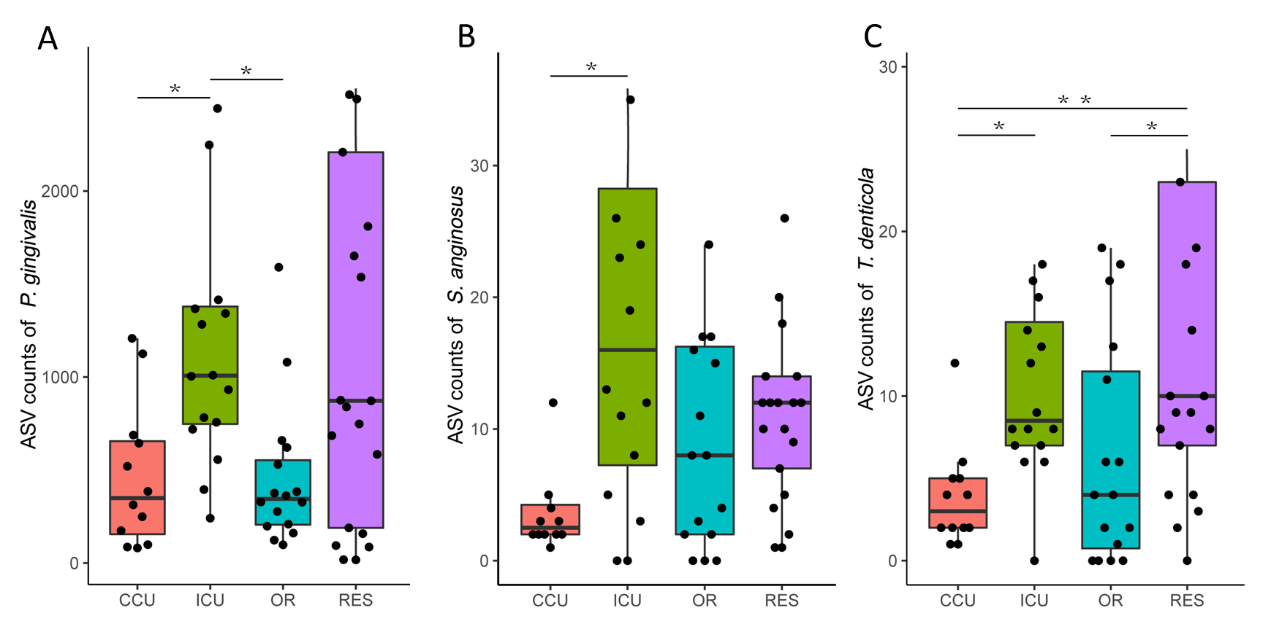


Fig. S6. Box chart showing the ASV counts of differential periodontal pathogens, including *P. gingivalis* (A), *S. anginosus* (B) and *T. denticola* (C). All three species showed low levels of OTU counts.


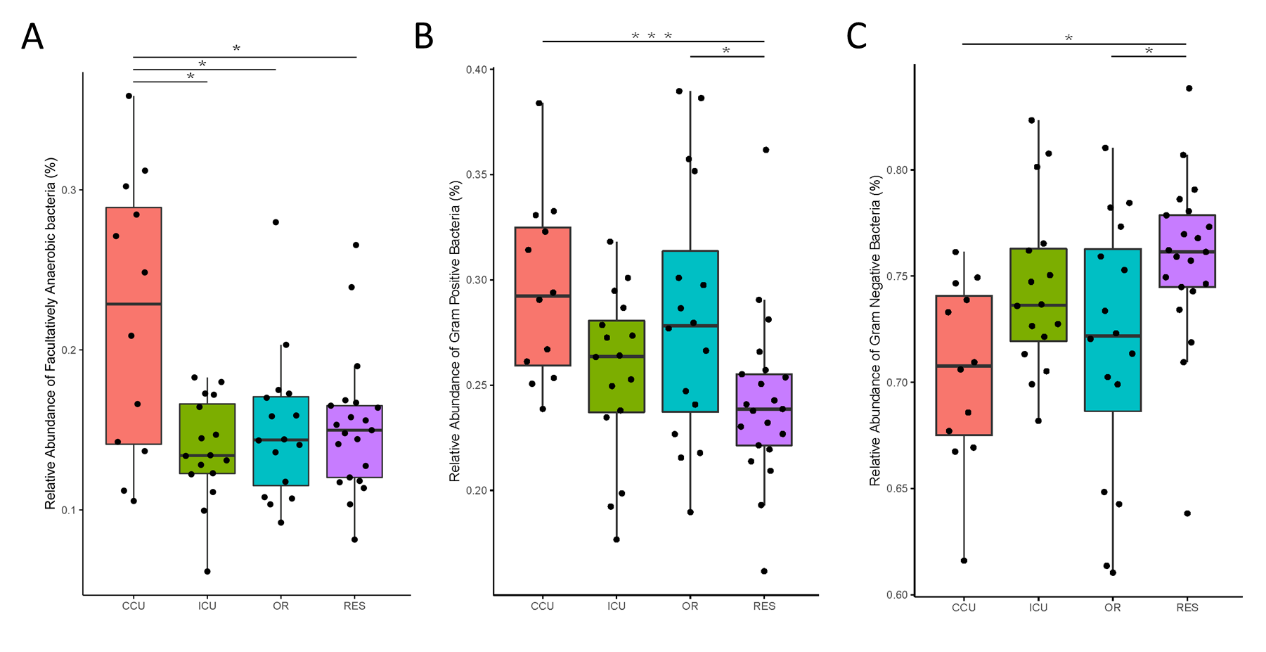


Fig. S7. Box chart showing differential predicted phenotypes, including facultatively anaerobic bacteria (A), gram positive bacteria (B) and gram negative bacteria (C) based on BugBase.
